# Efficacy and Safety of Low or Reduced Dose Direct Oral Anticoagulants Versus Dual Antiplatelet Therapy Following Left Atrial Appendage Closure: A Systematic Review and Meta-Analysis

**DOI:** 10.7759/cureus.69979

**Published:** 2024-09-23

**Authors:** Godfrey Tabowei, Anurag Rawat, Fayez S Alreshidi, Farook Ayyub Kantharia, Lubna Hanif, Hamzah M Alghzawi, Calvin R Wei, Neelum Ali

**Affiliations:** 1 Internal Medicine, Texas Tech University Health Sciences Center, Odessa, USA; 2 Interventional Cardiology, Himalayan Institute of Medical Sciences, Dehradun, IND; 3 Family Medicine, University of Hail, Hail, SAU; 4 General Surgery, University of Hail, Hail, SAU; 5 Family Medicine, Lancashire and South Cumbria NHS Foundation Trust, Preston, GBR; 6 Medicine, Karachi Medical and Dental College, Karachi, PAK; 7 Psychiatry, School of Nursing, Tennessee State University, Nashville, USA; 8 Research and Development, Shing Huei Group, Taipei, TWN; 9 Internal Medicine, University of Health Sciences, Lahore, PAK

**Keywords:** direct oral anticoagulants, dual antiplatelet therapy, left atrial appendage closure, systematic review and meta-analysis, thromboembolism

## Abstract

This meta-analysis aimed to compare the efficacy and safety of low-dose direct oral anticoagulants (DOACs) versus dual antiplatelet therapy (DAPT) in patients undergoing left atrial appendage closure (LAAC). A comprehensive literature search was conducted across multiple electronic databases, including PubMed, Embase, Cochrane Library, and Scopus, up to August 12, 2024. Studies comparing low-dose DOACs with DAPT in post-LAAC patients were included. The primary outcomes of interest were thromboembolic events, major bleeding, and all-cause mortality. Four studies, including two randomized controlled trials and two observational studies, met the inclusion criteria, with a total of 828 patients (319 in the DOAC group and 509 in the DAPT group). The meta-analysis revealed that patients treated with DOACs had a significantly lower risk of thromboembolic events compared to those receiving DAPT. DOACs were also associated with a significantly reduced risk of device-related thrombosis. Although the risk of stroke was lower in the DOAC group, the difference was not statistically significant. The risk of death did not differ significantly between the two groups. Regarding safety outcomes, patients receiving DOACs experienced fewer bleeding events compared to those on DAPT, with the difference being statistically significant. However, high heterogeneity was reported among the study results for bleeding events. These findings suggest that low-dose DOACs may be a more effective and safer alternative to DAPT for post-LAAC antithrombotic management, particularly in patients at high risk for both thromboembolic and bleeding events. DOACs demonstrated superior efficacy in reducing the risk of stroke, systemic embolism, and other thrombotic complications, while also minimizing bleeding risks.

## Introduction and background

For thromboprophylaxis, left atrial appendage closure (LAAC) has been routinely used in individuals with non-valvular atrial fibrillation (NVAF) [1]. The procedure aims to mechanically exclude the LAA, the major site of formation of thrombus in atrial fibrillation, thereby decreasing the risk of embolic stroke. While LAAC is an efficient substitute to oral anticoagulation, managing post-procedural antithrombotic therapy remains a clinical challenge [2-3]. Current guidelines recommend dual antiplatelet therapy (DAPT) with aspirin and clopidogrel for the initial phase following LAAC to prevent device-related thrombosis, followed by long-term single antiplatelet therapy [4]. But there is a chance that this approach will cause bleeding, especially in individuals who have had gastrointestinal bleeding in the past or are at high risk of bleeding [4].

The advent of direct oral anticoagulants (DOACs) has revolutionized the management of thromboembolic risk in AF, offering a safer and more convenient alternative to vitamin K antagonists (VKAs) [5]. DOACs have been shown to be at least as effective as VKAs in stroke prevention with a better safety profile, particularly concerning major bleeding events [6]. In recent years, there has been growing interest in exploring the role of low or reduced-dose DOACs as an alternative to DAPT following LAAC, especially in patients at high risk for both thromboembolic and bleeding events. Reduced-dose DOAC regimens could potentially offer a more favorable balance between thrombosis prevention and bleeding risk, but their efficacy and safety in this context remain uncertain [7-8].

Limited studies have compared low or reduced DOAC with DAPT in patients undergoing LAAC and these studies have enrolled less sample size. Given the variability in study designs, patient populations, and outcomes assessed, there is a need for a comprehensive synthesis of the existing evidence to guide clinical decision-making. This meta-analysis aims to systematically compare the efficacy and safety of low or reduced-dose DOACs versus DAPT in patients undergoing LAAC. The primary outcomes of interest include the incidence of thromboembolic events, major bleeding, and all-cause mortality. By integrating data from various studies, this analysis seeks to provide clarity on the optimal post-LAAC antithrombotic strategy, potentially informing future guidelines and clinical practice.

## Review

Methodology

Literature Search and Search Strategy

A comprehensive literature search was conducted to identify relevant studies comparing low or reduced-dose direct oral anticoagulants (DOACs) with dual antiplatelet therapy (DAPT) in patients undergoing left atrial appendage occlusion (LAAO). The search included articles published in electronic databases such as PubMed, Embase, Cochrane Library, and Scopus. The search strategy incorporated a combination of Medical Subject Headings (MeSH) terms and free-text keywords, including "left atrial appendage occlusion," "LAAC," "direct oral anticoagulants," "DOACs," "dual antiplatelet therapy," and "DAPT”. The search focused on studies published in English and included articles up until August 12, 2024. To find additional eligible studies, the reference lists of pertinent studies and review articles were also manually reviewed. Two authors conducted the search independently, and any disagreements between them were resolved through discussion.

Study Selection

The study selection process followed predefined inclusion and exclusion criteria. Studies were included if they: (1) involved patients undergoing LAAC, (2) compared low or reduced-dose DOACs with DAPT as post-procedural antithrombotic therapy, (3) reported on at least one of the outcomes of interest, including thromboembolic events, major bleeding, or all-cause mortality, and (4) were randomized controlled trials (RCTs), observational studies, or cohort studies. Exclusion criteria included studies that: (1) involved mixed patient populations without specific data on LAAC, (2) did not provide a direct comparison between low-dose DOACs and DAPT, or (3) were case reports, reviews, editorials, or abstracts without full-text availability. Two independent reviewers initially screened the titles and abstracts for eligibility, followed by a full-text review to ensure they met the inclusion criteria. Any discrepancies were settled through discussion, or by consulting a third reviewer.

Data Extraction

A standardized data extraction form was used to extract data from the chosen research. Study characteristics (authors, publication year, study design, sample size, etc.), patient demographics (age, gender, etc.), specifics of the intervention groups (type and dosage of DOACs, for example), and clinical outcomes (incidence of thromboembolic events, major bleeding, all-cause mortality) were among the data that were extracted. Additional data on study quality, follow-up duration, and funding sources were also collected. Two reviewers independently extracted the data, with any disagreements resolved by mutual agreement or by involving a third reviewer.

Data Analysis

The data collected were subjected to a meta-analysis, employing a random-effects model to account for potential variability among the studies. Pooled relative risk (RR) estimates, along with 95% confidence intervals (CIs), were calculated for each outcome of interest. Study heterogeneity was evaluated using the I² statistic, where an I² value exceeding 50% was indicative of significant heterogeneity. To assess the reliability of the results, sensitivity analyses were performed by excluding studies that exhibited a high risk of bias or presented outlying data. All statistical analyses were conducted using software such as Review Manager (RevMan) Version 5.4.1.

Results

**Figure 1:**
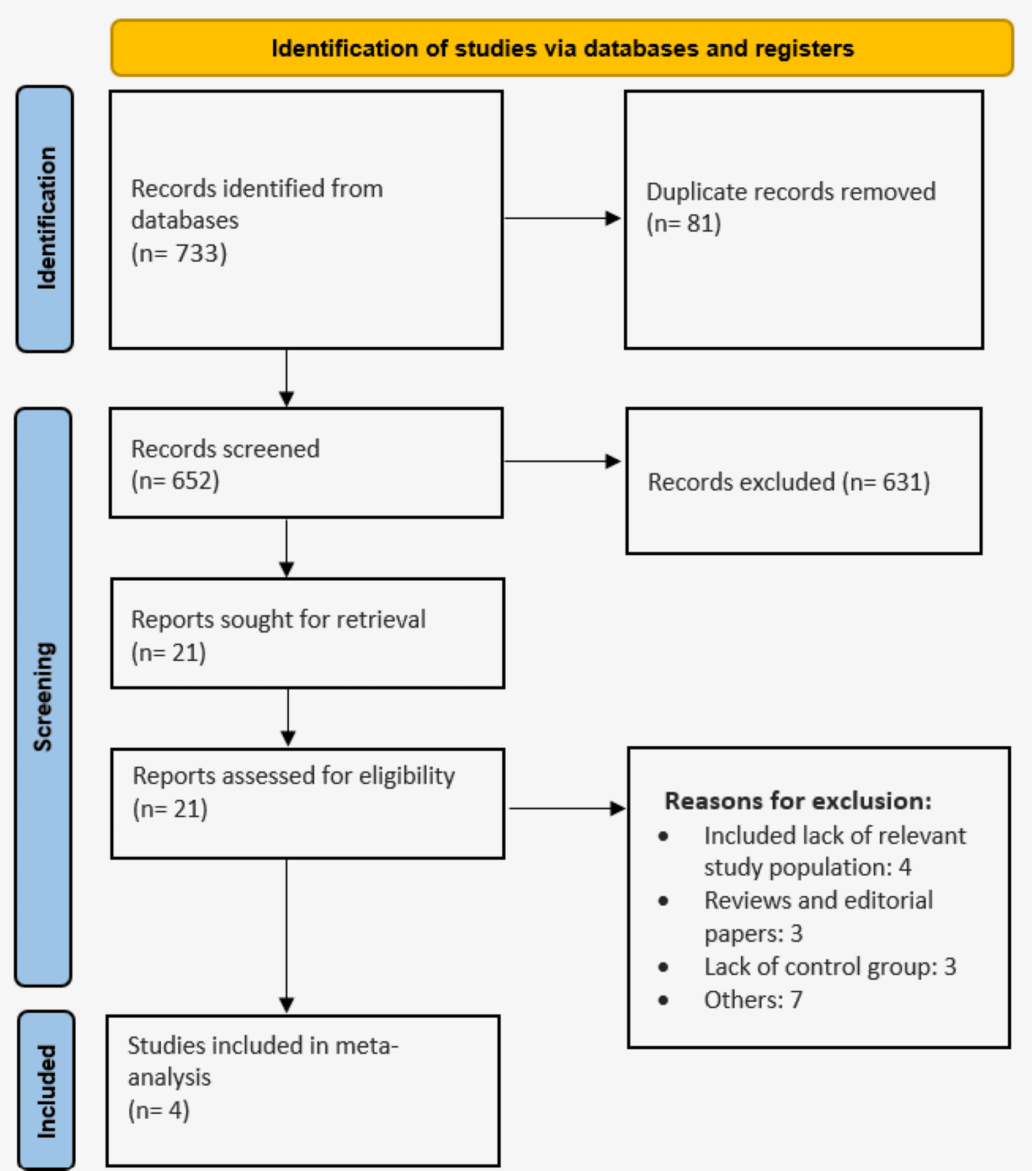
PRISMA flowchart (study selection process)

Through electronic search, we identified 733 studies. We removed duplicate studies and initial screening of 652 studies was done. We performed full-text screening of 21 studies based on predefined inclusion and exclusion criteria. Finally, four studies were included in this meta-analysis. Figure 1 shows the process of study selection. Table 1 presents the characteristics of the included studies. Out of four studies, two were non-RCT and two were RCTs. The pooled sample size in this study was 828 patients (319 in the DOAC group and 509 in the DAPT group). Table 2 and Table 3 present the quality assessment of non-RCTs and RCTs, respectively.

**Table 1:** Characteristics of included studies RCT: Randomized-control trial; DOAC: Direct oral anticoagulant; DAPT: Dual antiplatelet therapy

Author	Year	Design	Groups	Sample Size	Dose of DOAC	Follow-up Period	Age (Years)	Male (n)
Cepas-Guillen et al. [9]	2021	Non-RCT	DOAC	40	2.5mg/12h	3 Months	74.1	24
			DAPT	73			71.7	47
Della Rocca et al. [8]	2021	Non-RCT	DOAC	198	2.5mg/12h	13 Months	74.8	128
			DAPT	357			75.2	221
Duthoit et al. [10]	2020	RCT	DOAC	37	10 mg/24 hours	3 Months	78.4	20
			DAPT	33			77.5	23
Freixa et al. [11]	2024	RCT	DOAC	44	2.5mg/12h	3 Months	76.2	29
			DAPT	46			77.1	31

**Table 2:** Quality assessment of included studies (Non-RCTs) For Non-RCTs: New-castle Ottawa Scale was used.

Author ID	Selection of Participant	Comparability between Groups	Outcome and Exposure Assessment	Overall Quality Score			
Cepas-Guillen et al. [9]	3	2	3	8			
Della Rocca et al. [8]	4	2	2	8			

**Table 3:** Quality assessment of included studies (RCTs)

Author ID	Randomization	Concealment	Blinding of Participants and Personnel	Blinding of Outcome Assessor	Incomplete Outcome Data	Selective Reporting	Other Bias
Duthoit et al. [10]	No bias	Unclear	Bias	No bias	No bias	Unclear	No bias
Freixa et al. [11]	No bias	No bias	Bias	No bias	No bias	No bias	No bias

Meta-Analysis of Outcomes

**Figure 2:**
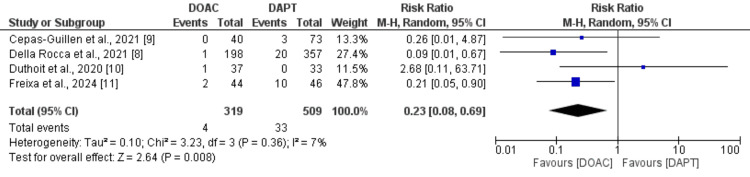
Comparison of primary composite outcome between two groups DOAC: Direct oral anticoagulant; DAPT: Dual antiplatelet therapy Sources: References [8-11]

Composite efficacy outcome: Figure 2 presents the findings from four studies that assessed the effectiveness of DOACs in preventing thromboembolic events and device-related thrombosis compared to DAPT. The combined analysis of these studies revealed a significantly lower risk of thromboembolic events in patients treated with DOACs compared to those receiving DAPT in the context of LOOC (RR: 0.23, 95% CI: 0.08 to 0.69, p-value: 0.03). The study results showed no significant heterogeneity.

Secondary efficacy outcomes: Table 4 summarizes the secondary efficacy outcomes, including the risks of device-related thrombosis, stroke, and death, in patients undergoing LOOC who were treated with either DOAC or DAPT. Patients receiving DOAC had a significantly lower risk of device-related thrombosis compared to those on DAPT (RR: 0.13, 95% CI: 0.02 to 0.67), with no significant heterogeneity observed among the study results. Although the risk of stroke was lower in the DOAC group compared to the DAPT group, the difference was not statistically significant (RR: 0.52, 95% CI: 0.08 to 3.26), and no significant heterogeneity was noted. Similarly, the risk of death did not differ significantly between the two groups (RR: 1.82, 95% CI: 0.27 to 12.47).

**Table 4:** Comparison of secondary outcomes between two groups DRT: Device-related thrombosis; RR: Risk ratio; CI: Confidence interval

Outcomes	RR	95% CI	I-Square
DRT	0.13	0.02 to 0.67	0%
Stroke	0.52	0.08 to 3.26	0%
All-cause Mortality	1.82	0.27 to 12.47	0%

**Figure 3:**
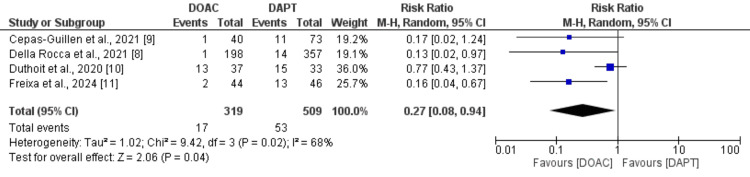
Comparison of bleeding events between two groups DOAC: Direct oral anticoagulant; DAPT: Dual antiplatelet therapy Sources: References [8-11]

Safety outcomes: Figure 3 presents the risk of bleeding events between patients receiving DOAC and DAPT. The number of bleeding events was lower in patients receiving DOAC (n=17) compared to DAPT (n=53) and the difference was statistically significant (RR: 0.27, 95% CI: 0.08 to 0.94). High heterogeneity was reported among the study results.

Discussion

In patients undergoing LOAC, DOACs have shown greater effectiveness than DAPT in preventing thromboembolic events while minimizing bleeding risks. For those undergoing LAAC, DOACs may serve as a viable option for patients suitable for short-term anticoagulation therapy. However, the evidence on DOAC efficacy and safety post-LAAC remains limited, and while a pooled analysis of existing studies could provide valuable insights for clinical practice, the current scarcity of studies makes it difficult to draw firm conclusions. This underscores the need for further research to determine the best anticoagulation approach after LAAC.

In the present meta-analysis, DOACs have demonstrated superior efficacy in reducing the risk of stroke, systemic embolism, and other thrombotic complications. This is largely attributed to their more predictable pharmacokinetic and pharmacodynamic profiles, as well as their targeted inhibition of specific coagulation factors, such as factor Xa or thrombin [12-13]. Unlike DAPT, which relies on the inhibition of platelet aggregation, DOACs directly target the coagulation cascade, thereby providing a more comprehensive and effective antithrombotic protection [14-15].

Due to substantial heterogeneity for each patient's unique bleeding risk and a dearth of randomized evidence, the best antithrombotic therapy following LAAO is not well established. In this regard, the current consensus statement on the best post-interventional antithrombotic medication regimen following LAAO suggests that the antithrombotic regimen be customized because most patients undergoing LAAO are at high risk for bleeding and there is insufficient data to support this recommendation [16].

In the main randomized clinical trials [17], warfarin was included in the antithrombotic regimen, starting with warfarin and aspirin (81 mg/day) for 45 days, then transitioning to aspirin (325 mg/day) and clopidogrel (75 mg/day) for six months, and finally continuing with aspirin (325 mg/day) alone. However, real-world observational studies have indicated that a three-month course of DAPT is the most frequently used antithrombotic approach following LAAO [18]. The choice of DAPT after LAAO is based on prior experiences with coronary stents and occlusion devices for the foramen ovale and atrial septum. Despite the ASAP study [19] showing that DAPT is associated with a reduced risk of stroke and device-related thrombosis (DRT), it does not fully mitigate the risk of major bleeding events after LAAO.

One of the most concerning side effects of LAAO treatment at the moment is device-related thrombosis, which affects at least 4% of patients after LAAO and is linked to a four- to five-fold rise in ischaemic events [20]. The current meta-analysis showed that patients on DOAC had a minimal incidence of thrombosis events attributable to the device. Our results, along with the available data, indicate that low- or reduced-dose DOAC after LAAC may provide Asian patients with NVAF with a good safety and effectiveness profile. It will take further randomized controlled trials to ascertain whether reduced-dose DOAC actually offers therapeutic benefits.

Study Limitations

The present meta-analysis has certain limitations. Firstly, only four studies were included in this meta-analysis. Out of these four studies, two were RCTs and two were observational. Therefore, studies are required in the future to confirm these findings. Secondly, we were not able to perform subgroup analysis on the basis of type of DOAC used and grouping variables. Thirdly, follow-up period of most of the included was less than six months. Future research should aim to expand the evidence base by conducting larger, well-designed randomized controlled trials with longer follow-up periods. Additionally, studies that evaluate the comparative efficacy and safety of different DOACs would provide valuable insights to guide clinical decision-making. Exploring the impact of DOACs on specific subgroups, such as patients with different comorbidities or undergoing different types of interventions, could also yield important information to optimize their use in various clinical scenarios.

## Conclusions

This meta-analysis demonstrates that low-dose direct oral anticoagulants (DOACs) are more effective than dual antiplatelet therapy (DAPT) in preventing thromboembolic events following left atrial appendage closure (LAAC). DOACs significantly reduced the risk of device-related thrombosis and overall thromboembolic events. Furthermore, DOACs showed a superior safety profile with fewer bleeding events. These findings suggest that DOACs may offer a promising alternative to DAPT for post-LAAC antithrombotic management, particularly in patients at high risk for both thromboembolic and bleeding events. However, the limited number of studies and short follow-up periods highlight the need for further research. Large-scale randomized controlled trials with extended follow-up are necessary to confirm these results and establish optimal antithrombotic management strategies post-LAAC.
